# Evaluation of Peripheral Blood Mononuclear Cell Processing and Analysis for Survival Motor Neuron Protein

**DOI:** 10.1371/journal.pone.0050763

**Published:** 2012-11-30

**Authors:** Dione T. Kobayashi, Douglas Decker, Phillip Zaworski, Karen Klott, Julie McGonigal, Nabil Ghazal, Laurel Sly, Brett Chung, James Vanderlugt, Karen S. Chen

**Affiliations:** 1 Spinal Muscular Atrophy Foundation, New York, New York, United States of America; 2 PharmOptima LLC, Portage, Michigan, United States of America; 3 Jasper Clinic, Kalamazoo, Michigan, United States of America; University of Edinburgh, United Kingdom

## Abstract

**Objectives:**

Survival Motor Neuron (SMN) protein levels may become key pharmacodynamic (PD) markers in spinal muscular atrophy (SMA) clinical trials. SMN protein in peripheral blood mononuclear cells (PBMCs) can be quantified for trials using an enzyme-linked immunosorbent assay (ELISA). We developed protocols to collect, process, store and analyze these samples in a standardized manner for SMA clinical studies, and to understand the impact of age and intraindividual variability over time on PBMC SMN signal.

**Methods:**

Several variables affecting SMN protein signal were evaluated using an ELISA. Samples were from healthy adults, adult with respiratory infections, SMA patients, and adult SMA carriers.

**Results:**

Delaying PBMCs processing by 45 min, 2 hr or 24 hr after collection or isolation allows sensitive detection of SMN levels and high cell viability (>90%). SMN levels from PBMCs isolated by EDTA tubes/Lymphoprep gradient are stable with processing delays and have greater signal compared to CPT-collected samples. SMN signal in healthy individuals varies up to 8x when collected at intervals up to 1 month. SMN signals from individuals with respiratory infections show 3–5x changes, driven largely by the CD14 fraction. SMN signal in PBMC frozen lysates are relatively stable for up to 6 months. Cross-sectional analysis of PBMCs from SMA patients and carriers suggest SMN protein levels decline with age.

**Conclusions:**

The sources of SMN signal variability in PBMCs need to be considered in the design and of SMA clinical trials, and interpreted in light of recent medical history. Improved normalization to DNA or PBMC subcellular fractions may mitigate signal variability and should be explored in SMA patients.

## Introduction

Spinal Muscular Atrophy (SMA) is a progressive and largely pediatric neuromuscular disease that is the primary genetic cause of death in infants and toddlers. SMA manifests with profound proximal muscle weakness due to the degeneration of anterior horn motor neurons in the spinal cord [1], [2]. While most patients born with SMA never sit independently and have been previously reported to die by age 2 (Type 1), these patients are now living longer due to improvements in the standard of care [3]–[6]. Patients with milder forms of SMA are able to sit (Type 2), walk (Type 3), and have normal or near normal lifespans – thus the majority of patients are adolescents or older [3]–[5], [7]. SMA is the epitome of a disease with a highly unmet medical need: it is ultimately a terminal disease for many Type 1 patients, Type 2 and 3 patients over time experience progressive loss of motor function and skeletomuscular deformities that impact breathing, and there is no effective treatment for any form of the disease. SMA is genetically unique, as the disease is caused by loss of the Survival of Motor Neuron 1 (SMN1) gene but a near perfect phenocopy gene (SMN2), can modify disease phenotype and partially compensate for the loss of functional SMN protein. Patients with milder forms of disease generally have more copies of the SMN2 gene [8].

Due to the theoretical strength of the biological rationale for SMN as a therapeutic target for SMA, several groups are exploring a multitude of approaches for SMN upregulation. These efforts include a number of programs for novel antisense oligonucleotides, gene therapy vectors, and small molecules targeting SMN that are at different stages of preclinical and clinical drug development [9]–[19]. Trials that feature drug candidates that systemically increase SMN will benefit greatly from having a pharmacodynamic marker to gauge target engagement and dose selection. However, though SMA is a disease of the spinal cord or perhaps the motor circuit, these tissues are not accessible for sampling in trials. Moreover, trials will take place in a pediatric population, further limiting the kind, scope, and volume of sampling possible for biomarkers. The ideal biomarker would provide a readout on SMN in a peripherally accessible cell population that can act as a surrogate for SMN changes in target tissues. Several SMA researchers have previously published on the use of blood cells for the purpose of evaluating SMN protein, and there are existing collections of these samples from SMA patients [8], [20]–[26]. In addition there is extensive literature on optimizing collection, processing, and storage protocols for peripheral blood mononuclear cells (PBMCs) from immunology and other fields [27]–[29]. Using a commercially available assay for measuring SMN protein, we interrogated PBMCs as a matrix for peripheral SMN analysis, focusing on testing a number of variables that impact matrix and signal stability and variability. After a series of studies with freshly collected blood samples from healthy adults, SMA patients and carriers, we have developed protocol that enables reliable use of PBMCs for SMN signals in multi-centered clinical trials [30].

## Results

### Studies 1 and 2: Impact of Processing Delays on SMN Signal and PBMC Yields

**Figure 1:**
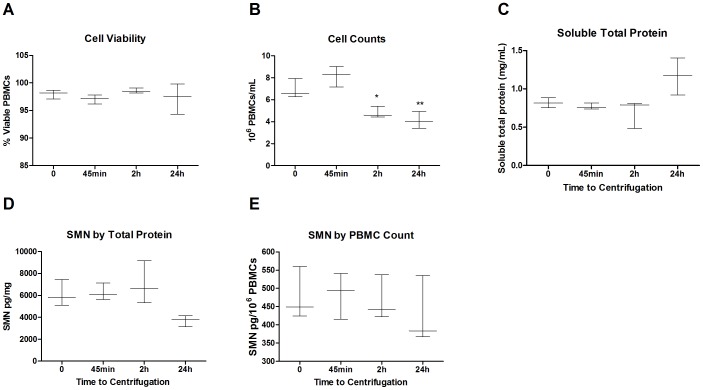
Study 1: Impact of short-term PBMC processing delays. PBMCs were collected via CPT tubes from 3 individuals and processed with delays of 0, 45 minutes, 2 h and 24 h prior PBMC isolation by centrifugation. **A:** Cell viability was similar at all timepoints for all subjects, ranging from 94–99%. **B:** Cell counts were consistent through 45 minutes, but were significantly reduced by 30–40% with delays of 2 h and 24 h compared to the 0 h timepoint. **C:** Total soluble protein was consistent with up to 2 h processing delays, however at 24 h there was a trend towards increased protein concentrations by up to 40%. **D**: SMN levels with 24 h processing delays were accordingly reduced when normalized by total protein. **E**: SMN levels by cell counts were similar with all delays examined, albeit with trends for higher variability than the SMN signal generated by protein normalization. In Figure 1 error bars indicate the minimum and maximum values while the horizontal bar indicates the median value.

In Study 1 whole blood was collected from three healthy individuals in CPT tubes and processed to PBMCs immediately or with delays prior to PBMC isolation of 45 min, 2 h, or 24 h at room temperature. All samples were processed to lysates immediately after PBMC isolation. While viable cell numbers did not decrease with processing delays, the number of PBMCs isolated with 2 h and 24 h processing delays declined by ∼30% compared to immediately processed samples (Figure 1A–B). Total soluble protein was stable through 2 h; however, it tended to increase by 24 h (Figure 1C). Given that SMN protein signal can be calculated by total protein normalization, PBMC SMN signal appears to decline when isolated from CPT tubes with a 24 h delay (Figure 1D). However, when normalizing by cell counts the SMN signal is equivalent for all time delays (Figure 1E).

**Figure 2:**
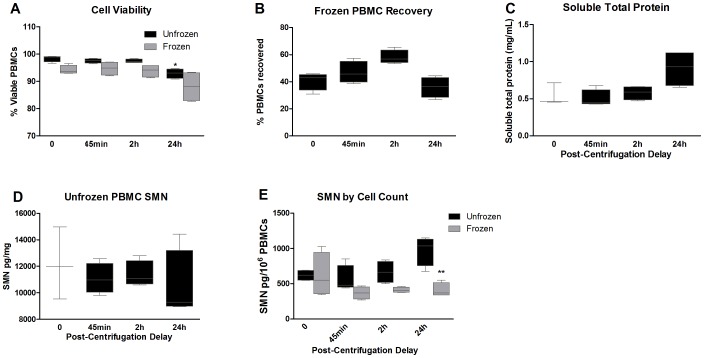
Study 2: Impact of post-isolation delay and cell freezing. SMN signals were evaluated in PBMCs from 4 individuals that were analyzed with 45 minute, 2 h and 24 h delays after cell isolation. A subsample from each timepoint was frozen to assess post-freezing viability and SMN signals. **A:** Cell viability was generally lower in PBMCs that had been frozen, ranging from 88–95% viability compared to 93–98% in unfrozen cells. Statistical comparisons were made to the 0 h timepoint. **B**: The comparative recovery of viable PBMCs after freezing relative to fresh samples was only ∼40–60% at all timepoints, suggesting a major loss of cells in the freezing process. **C**: Delaying the processing of isolated PBMCs to lysates had no impact on protein concentrations through delays of 2 h, however there was again a trend for increased protein concentrations in samples left for 24 h. **D**: SMN levels (normalized to protein concentrations) in unfrozen PBMCs were generally similar across all timepoints, despite wide variability in signals. **E**: Analysis of SMN by cell counts revealed that SMN signals in unfrozen cells tended to increase with post-isolation delays. Frozen cell SMN signals generally seemed to decrease over time compared to both frozen cells processed with minimal delays or compared to unfrozen cells. Signals from frozen cells with 24 h post-isolation delays were lower than unfrozen cells. Error bars represent minimum and maximum values. In Figure 2 the bodies of the boxplots indicate the first and third quartiles, while the horizontal bar indicates the median.

Study 2 was conducted to explore the simulated impact of isolating PBMCs at a clinical collection site and shipping them overnight to a central site for further processing. Four healthy individuals (three from Study 1) provided blood samples for PBMC isolation by CPT tubes. Cells were isolated immediately or with delays of 45 min, 2 h or 24 h prior to PBMC lysis and SMN extraction. Parallel samples were also frozen at −80°C to examine the recoverability of cells and SMN signal after freezing. Cell viability was overall good, ranging from 90–98%, and was modestly reduced in unfrozen PBMCs at 24 h (Figure 2A). Delaying the processing to lysates after cell isolation did not alter cell counts (data not shown). When comparing the cell counts recovered from frozen PBMCs to that of unfrozen PBMCs processed with no delays, there was a dramatic 45–60% drop in comparative viable cell counts. Total protein as measured in the unfrozen PBMCs with various processing delays again showed a tendency towards increasing at 24 h (Figure 2C). SMN levels in frozen cells either normalized by protein content or cell count did not differ with post-isolation processing delays. However, frozen cells showed a 34% reduction (p<0.01) in signal compared to the unfrozen cells with 24 h delays (Figure 2D–E).

### Study 3: Comparison of PBMC Isolation Methods

**Figure 3:**
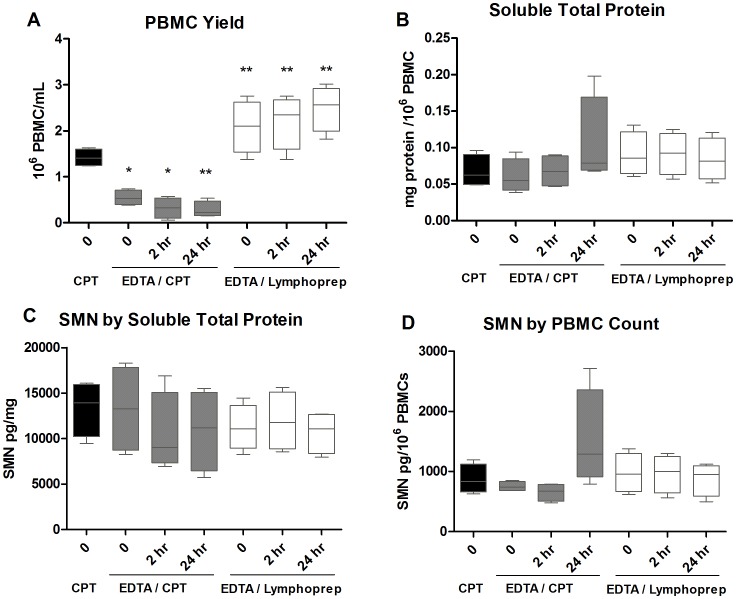
Study 3: Comparison of PBMC isolation methods and SMN measures from various blood fractions. Due to the variability in SMN signal and protein concentrations seen with CPT tube PBMC isolation, other methods were explored using the four subjects from Study 2. **A**: PBMC yield was greatest in samples collected with EDTA tubes and subsequent Lymphoprep gradient separation, and showed no statistically significant changes with isolation delays of up to 24 h. **B**: Total soluble protein tended to increase with 24 h delays in samples collected by EDTA tube and isolated by CPT tubes, while there was no obvious change in protein concentrations in EDTA/Lymphoprep processing. **C**: SMN as measured by total protein tended to decrease with isolation delays with EDTA/CPT processing. SMN signals were similar with delays up to 24 h with EDTA/Lymphoprep processing. **D**: SMN by PBMC counts was variable for both EDTA/CPT and EDTA/Lymphoprep processing methods. However, the EDTA/Lymphoprep values were generally overlapping and did not appear to decrease from the no-delay timepoint (t = 0). In Figure 3 body of the boxplots indicate the first and third quartiles, while the horizontal bar indicates the median.

Due to the dramatic loss of cells with freezing and the tendency for total protein increasing after 24 h processing delays with CPT tubes, we investigated cell yields and SMN signals with isolation over resin gradients (Lymphoprep). Blood from 4 individuals was collected into CPT or EDTA tubes. Samples collected by EDTA tubes were processed by CPT tube or by Lymphoprep gradient. Separate samples were also collected and processed in CPT tubes. PBMCs were collected in EDTA and isolated by CPT or Lymphoprep with no delays or with 2 h and 24 h delays before being processed immediately to lysates. PBMC yield was greatest in the EDTA collected and Lymphoprep isolated samples which were on average 1.5–4.5x greater than that of any CPT-processed samples at 0 h (Figure 3A). EDTA collected samples isolated by CPT also had a tendency to increase in total protein with 24 h delays, while there was no change in Lymphoprep isolated samples with 2 h or 24 h delays (Figure 2B). Overall SMN signal with Lymphoprep processing had similar ranges at all timepoints with coefficients of variance (CVs) that ranged from 3–11% for SMN normalized by total protein and 11–16% for SMN normalized by cell count (Figure 3C–D). CPT processed samples had signals that trended lower and greater CVs from 11–32% and 24–80% for SMN normalized by total protein and cell counts. (Figure 3C–D). Also, samples from the same individuals with Lymphoprep processing had less variable SMN levels by protein or cell count normalization than those of the same subjects’ samples processed by CPT with EDTA collection (Figure S1). Lymphoprep isolation was used in subsequent experiments, due to robust PBMC yield, and reduced variability with processing delays compared to CPT processed samples (F = 0.49 versus 4.3 respectively).

### Analysis of Blood Components for SMN Signal

Using samples collected with EDTA, whole blood and blood pellets were assessed for SMN signal, as they are more straightforward samples to process than PBMCs (Table 1). Protein quantitation was not performed due to the red color of the samples. Using dilutions between 1∶2 and 1∶8, there was significantly more interference in the matrices at lower dilutions that could not be completely eradicated in all subjects even at 1∶8. In a repeat experiment using the same materials at higher dilutions, several samples at 1∶10 and 1∶20 dilutions were below the limit of detection and signals were 5–20x lower than previous signals at 1∶5, suggesting matrix instability.

**Table 1:** Dilution adjusted SMN Signal in whole blood and blood pellets (pg/mL blood).

		Blood Pellet			Whole Blood			
**Subject**	**Neat**	**1∶2**	**1∶4**	**1∶8**	**Neat**	**1∶2**	**1∶4**	**1∶8**
**1002**	569	1550	2108	2008	695	1894	2576	2454
**1012**	561	1636	1996	1784	686	2000	2440	2180
**1013**	1738	4236	5564	5992	2124	5177	6800	7324
**1032**	619	1932	2520	2392	757	2361	3080	2924

Whole blood and red blood cell pellets were evaluated for SMN signals with the four individuals from Study 3. SMN protein was detectable in both matrices. Dilutional linearity was not observed across all samples or at the same dilutions across samples, suggesting there was biological interference in blood pellet and whole blood.

### Optimizing Cell Densities and Reagents for Lysis and SMN Extraction

**Figure 4:**
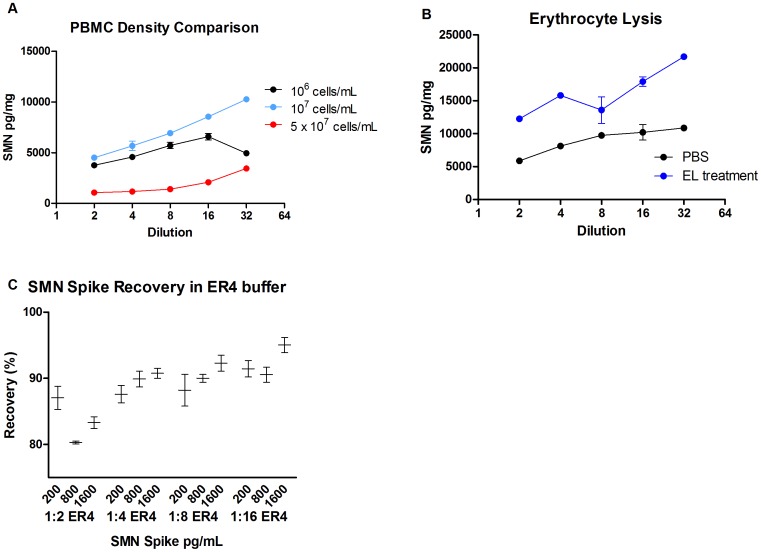
Optimizing cell densities and reagents for lysis and SMN extraction. To examine the impact of lysates and lysis reagents on SMN signal a number of factors were tested. **A**: SMN signal was evaluated in lysates created from a single sample purchased from AllCells. PBMCs were processed in ER4 at densities of 10^6^, 10^7^ and 5×10^7^ cells/mL and the resulting lysates diluted from 1∶2 to 1∶32 for analysis in the SMN ELISA. At the highest concentration SMN signal was linear with adjusted dilutions until after 1∶16, suggesting interference at this density. Concentrations of 10^6^ and 10^7^ cells/mL had parallel linear increases in signal between 1∶2 and 1∶8, with the 10^7^ dilution continuing to rise at the 1∶32 dilution. **B**: Treatment of PBMCs in erythrocyte buffer for 5 minutes increased the SMN signal by roughly twofold over the untreated cells. **C**: SMN protein standard was formulated in different dilutions of ER4 at different concentrations to determine the impact on spiked SMN signal recovery. Use of 1∶4 to 1∶16 ER4 reagent allowed for >90% signal recovery across several SMN levels. Error bars represent standard deviations in Figures 4A–B. In Figure 4C error bars indicate the minimum and maximum values while the horizontal bar indicates the median value.

To evaluate the impact of cell density on optimizing SMN protein signal, different densities of cells 10^6^, 10^7^ and 5×10^7^ PBMCs were analyzed in ER4 reagent dilutions from 1∶2 to 1∶32 dilutions (Figure 4A). Changing cell densities measurably affected SMN signal. The 5×10^7^ cell density produced the lowest levels of SMN signal of the three cell densities by 2–3x, ranging from 1073–3448 pg/mg SMN, suggesting that SMN extraction or cell lysis is inefficient in higher cell concentrations. Densities of 10^6^ and 10^7^ cells/mL produced SMN signals ranging from 3766–6574 and 4508–10254 pg/mg SMN respectively. Given that the 10^7^ cell/mL concentration was linear at all concentrations and had the highest signal of the three, the ER4 dilution of 1∶8 was chosen for all subsequent experiments.

Given the possibility of matrix interference on SMN signal due to red blood cells, the effects of erythrocyte lysis (EL) were examined. PBMCs isolated by EDTA/Lymphoprep were split into two samples, one for processing with and one without EL (Figure 4B). EL treatment increased SMN signal relative to PBS treatment by 1.5–2x across ER4 dilutions of 1∶2 to 1∶16. EL treatment became standard for all subsequent experiments.

As all the PBMC samples will retain some ER4 as a result of their processing and ER4 increased SMN signal in prior studies [30], we sought to also use ER4 in the SMN protein standard to ensure equivalent quantification of SMN signals. SMN standard was diluted in the presence of 1∶2 to 1∶32 ER4 in assay buffer, and recovery of SMN protein spikes of 200, 800, 1600 pg/mL were quantified with recovery signal expressed as a percentage of total spike amount (Figure 4C). At all dilutions recovery of the SMN spike was greater than 80%, with recovery at 88–93% for dilutions at 1∶4 to 1∶16, including the 1∶8 ER4 dilution that had performed well in PBMC density and EL processing analyses. CVs at all dilutions and spike concentrations ranged from <1–3%. In all subsequent experiments ER4 was used at a dilution of 1∶8 in both cell lysates and SMN standard. The optimized protocol for SMN protein analysis in PBMCs by ELISA is available here at the TREAT-NMD website: http://www.treat-nmd.eu/downloads/file/sops/sma/SMA_M.1.2.005.pdf.

### Study 4: Inter- and Intra-individual SMN Signal Variability

Using the optimized PBMC protocol, blood from six healthy adults was collected at different timepoints for SMN protein evaluation. Blood was collected at t = 0, 6 h, 24 h, 7d, and 30d. At t = 3 h subjects were fed a meal high in carbohydrate content to explore the impact of meals on SMN signal. At the 7d timepoint blood collected from individuals were fractionated to CD4, CD8, CD14, CD19, and CD56 PBMC subtype populations using a Miltenyi positive selection bead system.

**Figure 5:**
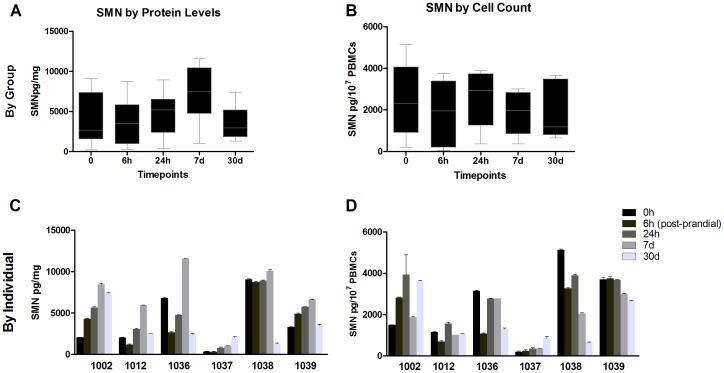
Study 4: Inter- and intra-individual SMN signal variability. While PBMC SMN levels normalized by total protein levels or cell counts are similar within a group; measures of individuals across timepoints reveal large differences. The 6 h timepoint is 3 h post-prandial to a large carbohydrate rich meal. **A**: PBMC SMN by protein level are not different by group analysis from at T = 0 h through 30d after initial collection. **B**: PBMC SMN by cell counts is not different by group analysis. **C,D**: SMN protein levels in PBMCs normalized by protein or cell counts by individual vary up to 8x over multiple blood collection timepoints, and between individuals the SMN levels can diverge by >25x. In Figures 5A–B the bodies of the boxplots indicate the first and third quartiles, while the horizontal bar indicates the median. In Figures 5C–D error bars depict standard deviation.

**Figure 6:**
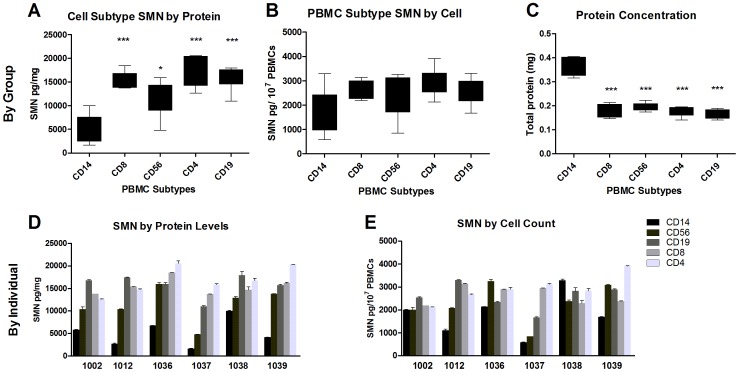
Study 4: Subcellular PBMC populations and SMN signal. SMN levels were analyzed by CD+ cell subtype at the 7d timepoint from Study 4. **A**: Analysis of SMN by PBMC cell subpopulation revealed that the CD14+ population had statistically significant reductions in SMN levels. **B**: Fractionation of PBMCs with normalization by cell count showed no differences in SMN signal in group analysis. **C**: Evaluation of total soluble protein levels by CD+ population revealed that CD14+ cells had double the protein concentrations of CD4+, CD8+, CD19+, and CD56+ cells. This differential is sufficient to drive variability in situations that cause CD14+ populations to fluctuate. **D**: SMN levels (normalized by protein) show consistently lower levels in CD14+ fractions compared to all other fractions, with differences up to 7x within individual PBMC subpopulations. **E**: SMN in individuals as measured by cell counts were also variable ranging up to 3.5x between individuals’ subcellular populations, but was overall less variable than protein normalized SMN measures. In Figures 5A–C the bodies of the boxplots indicate the first and third quartiles, while the horizontal bar indicates the median. In Figures 5D–E error bars depict standard deviation.

SMN levels overall were not significantly different between timepoints for the entire set of subjects (Figures 5A–B). However, on an individual basis SMN at the same timepoints varied highly whether the signal was normalized to total protein (up to 37x) or cell count (up to 20x), and the profiles of SMN over time were distinct between subjects (Figures 5C–D). Within individuals, SMN values also varied over time, from 2x to 10x with CVs for SMN by protein ranging between 30–62%, and for SMN by cell count by 9–65%. There was no clear general pattern of increase or decrease in SMN between 0 h and the other timepoints (all F<1) (Figure 5C–D). The overall SMN signals between the 24 h and 7d timepoints were generally similar among the subjects by cell counts (Figure 5D) but more variable by 30d. Subtype fractionation at 7d revealed that while SMN signals normalized by cell count were similar between most cell populations, the CD14+ cell population SMN levels were half that of other cell types (p<0.001, Figure 6A). These results were due to a difference in the basis for normalization, as the CD14+ protein concentrations were double that other subtypes (Figure 6C). There were no marked differences in the SMN levels normalized by cell count compared to other populations, but there appeared to be greater variability in the CD14+ and CD56+ populations that contained monocytes, macrophages and killer T-cells. About 40–70% of all PBMCs were quantified by measuring the various CD+ cell populations in Study 4 (data not shown).

### Study 5: Impact of Respiratory Infections on SMN Signal

**Figure 7:**
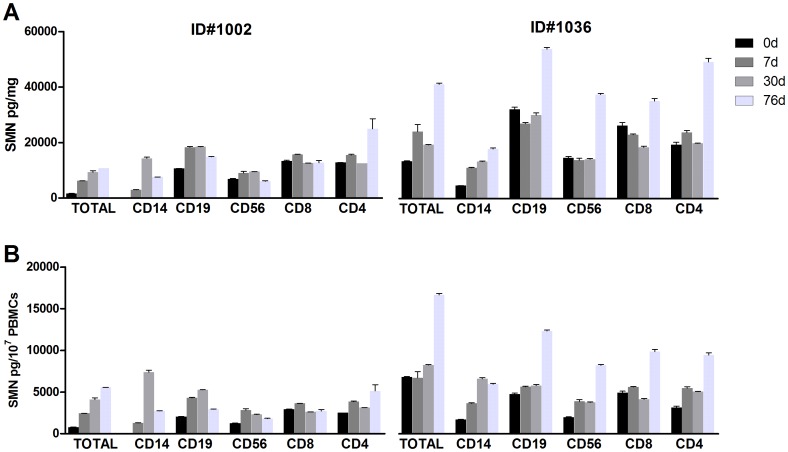
Study 5: Immune modulation of PBMC SMN signal. SMN levels were examined in 2 Study 4 individuals that became symptomatic with upper respiratory infections. **A**: PBMCs were fractionated for SMN protein analysis with total protein normalization. The CD14+ population was highly variable, ranging up to 4–70x in subjects 1036 and 1002 over time. **B**: The SMN signal in CD14+ populations was also highly variable when quantified by cell counts ranging from 2–140x. By both protein and cell quantification method, SMN signal appeared to be least variable in the CD8+ subpopulation. In Figure 7 error bars represent standard deviations.

To further investigate the variability of SMN signal in PBMCs due to cell subtypes, blood from two subjects was collected for further fractionation analysis. Blood from subjects 1002 and 1036 was collected 2–3 days after the onset of infection symptoms (about 5 weeks after the 30d collection in Study 4). Subjects presented with fevers of >38.3°C 2–3 days before blood draws and were afebrile with sore throat, cough, congestion and headache at t = 0. Subject 1002 was asymptomatic at the 30d collection and Subject 1036 had a suspected sinus infection and allergies at 30d. By 76d both subjects were asymptomatic. SMN levels in the CD14+ population during symptomatic respiratory illness were 4–35x lower than at asymptomatic timepoints with CVs ranging from 47–121% (Figure 7A–B), and with cell counts drove down the total SMN levels (Table S1). Regardless of the normalization technique used, the CD8+ population had the least amount of SMN signal flux between the two subjects, ranging from 0.1–2x across timepoints (CVs ranging from 4–42%). Overall the range of SMN levels from the subject 1002 in across studies was relatively similar, with 0 h-30d SMN protein counts for Study 4 ranging from ∼2500–8000 pg/mg and ranging from 2000–12000pg/mg for 0 h to 76d for Study 5. Subject 1036 had wider variations in SMN levels between the two studies, starting with 8000–2500 pg/mg SMN for 0 h and 30d in Study 4, but quintupling to 40000 pg/mg by 76d in Study 5. Given the continued symptoms experienced by this subject over a 16 week period, it is unclear whether Subject 1036’s high 76d SMN measurement is a due to chronic elevation following a period of illness or a sign of impending new infection.

SMN measurements made by DNA quantification (Cyquant assay) and cell counts determined by hemocytometer were compared with cells from the 76d timepoint for subject 1002. Cells were diluted 2-fold from 3.1×10^3^–10^6^ cells. Values were highly correlated and the overall R^2^ = 0.99 between the cell numbers/mL and the Cyquant assay DNA content fluorescent units (data not shown).

### SMAF-001: SMN in SMA Patients and Carriers

Using the optimized methods developed in prior experiments, SMN was measured across a population of self-identified SMA patients and their carrier parents. Individuals were enrolled into the study over an 8 week period, and clinical and medical history data and blood samples were collected from a single visit from SMA Type 2 (N = 7) and Type 3 (N = 5) patients aged 1–60 years and carriers aged 25–57 years (N = 15) (Tables 2–3). Although the study protocol allowed for healthy pediatric subjects, none were enrolled. Samples were collected and PBMCs isolated within 24 h. Samples were processed to lysates and analyzed in the ELISA after storage for 48 h, 1 mo, 3 mo and 6 mo to evaluate sample stability and comparability of values over time.

**Table 2:** Study SMAF-001 demographics.

Characteristics	N	Age (years)
**SMA Type 2**	7	1–50
**Male**	1	1.4
**Female**	6	1–50
**SMA Type 3**	5	2–60
**Male**	4	2–60
**Female**	1	4
**Carriers**	15	25–57
**Male**	6	32–48
**Female**	9	25–57

SMA Type and Carrier status was self-reported by subjects or their consenting parents. Subjects identified as Type 2/3 were included in the Type 3 group.

**Table 3:** SMAF-001 SMA patient characteristics.

Subject	Type	Current Function	Highest Function	Age (years)	Onset (Years)
3002	2	Ambulatory	Walking	4	0.9
3004*	2	Ambulatory	Walking	2	0
3005	2	Non-ambulatory	Sitting	50	3
3007	2	Ambulatory	Walking	3	1.3
3017	2	Non-ambulatory	Sitting	22	0.6
3026*	2	Non-ambulatory	Standing	4	0.06
3012	2	Non-ambulatory	Sitting	1.4	1.1
3008**	3	Non-ambulatory	Sitting	60	15
3025	3	Ambulatory	Walking	3	0.6
3021	3	Non-ambulatory	Walking	13	1.5
3028	2/3	Ambulatory	Walking	4	3
3029*	2/3	Ambulatory	Walking	2	2

* Subject 3004 is the sibling of Subject 3002, Subject 3026 is the sibling of Subject 3025, and Subject 3029 is the sibling of Subject 3028.

** Subject 3008 was not diagnosed until age 50.

**Figure 8:**
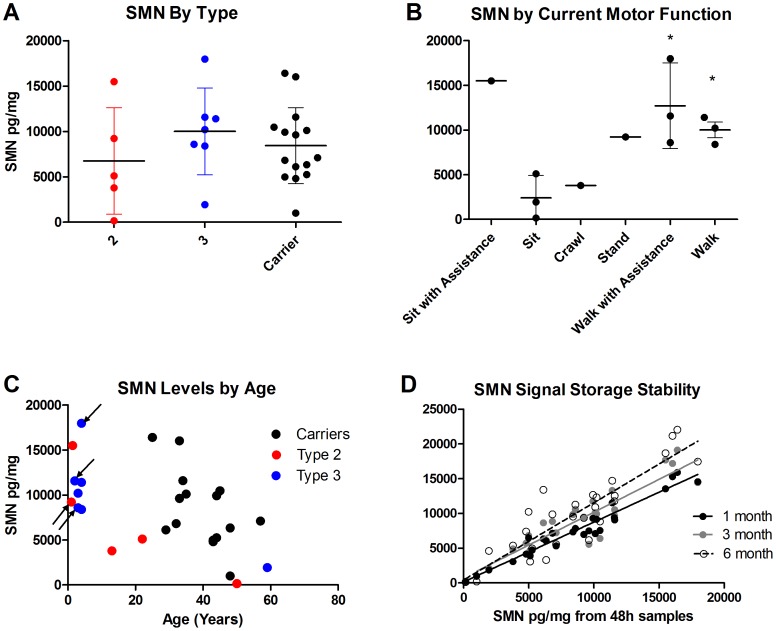
Study SMAF-001: SMN in SMA patients and carriers. SMN protein (normalized by total protein) was evaluated in Type 2 and 3 SMA patients and Carriers for differences by motor function and stability of signal with lysate storage. **A**: SMN protein classified by Type and Carrier status completely overlapped between groups. When adjusted by age there was a significant difference in SMN between patients and adult carriers (p<0.001) but not between SMA Types (p = 0.75). **B**: SMN levels differentiated by current motor function appeared to distinguish between sitters and ambulatory patients (P<0.05), with the exception of a 1.4 year old recently diagnosed child who could sit with assistance. When controlling for age this trend was not statistically significant (p = 0.97). **C**: SMN levels were plotted against subject age and there was a trend towards lower SMN levels in older individuals. The correlations for age-related decline were different between SMA and Carriers, with R^2^-values at 0.65 (p<0.005) and 0.30 (p<0.05) respectively. Arrows depict subjects taking valproic acid, a drug with putative SMN-upregulating effects. **D**: Samples from each subject were processed and frozen as lysates for storage for 48 h, 1 month, 3 months, and 6 months. Comparability of signals between the 48 h timepoint and subsequent timepoints was generally high, with R^2^-values of 0.79–0.94 for each timepoints. In Figure 8A–B error bars indicate the minimum and maximum values while the horizontal bar indicates the median value. In Figure 8D the trendlines depict R^2^-values.

SMN analysis between SMA Types and carriers revealed significant overlaps between them, but when adjusted by age in regression analysis, SMA patients and Carriers do indeed have significantly different SMN levels largely due to their difference in group age ranges (<0.001) (Figures 8A, S2A). Even when adjusting for age there were no differences between SMA Types’ SMN levels. When patient SMN levels were separated by current level of function, differences between groups seemed more apparent; however there were no significant differences when controlling for age (Figure 8B, S2B). There was a trend towards lower SMN levels over age regardless of Type or carrier status (Type 2 R^2^ = 0.63, Type 3 R^2^ = 0.87, carrier R^2^ = 0.29, p = 0.03). Patients taking valproic acid or valproic acid and sodium phenylbutyrate had SMN levels that were overlapping with individuals of the same general age (Figure 8C). Decreases in SMN signal in samples stored as frozen lysates for 1–6 mo after initial blood collection ranged from 9–17% for all samples across all timepoints (data not shown). Overall comparability of SMN values in samples from the same subjects was high, with R^2^ values of 0.94, 0.82, and 0.79 at 1 mo, 3 mo and 6 mo respectively (all p<0.001) (Figure 8D).

PBMC counts were overall significantly higher in SMA patients than in carriers (p<0.05, with mean of 1.7×10^6^ versus 5.7×10^6^ PBMCs/mL respectively) (Figure S3). While there was no correlated association of PBMC counts with age, 4 subjects in the younger SMA patient group were taking putative SMN-upregulating drugs, which could potentially have some effect on SMN (Figure 8C, arrows). All patients 13 years and older took at least 2 prescription drugs indicated for acid reflux, allergies, anxiety, high blood pressure, cholesterol control, insulin control, and/or osteoporosis. Some of these medications have been reported to have effects on blood including white blood cells. PBMC isolation was problematic in two Study SMAF-001 samples, with low PBMC counts in one and visible red blood cell contamination at the PBMC layer in the other. The first individual was taking Atenolol, which can reduce white blood cell counts, and the other subject was taking Flonase and Singulair.

## Discussion

The experiments reported here examine the factors affecting SMN protein signal variability in a peripherally accessible cell matrix that is expected to be a critical pharmacodynamic biomarker for imminent or ongoing drug trials for SMA [15], [31], [32]. PBMCs have been harvested and utilized effectively to evaluate target engagement and cellular efficacy in numerous international multi-center clinical trials, most notably in the human immunodeficiency virus (HIV) field [27]–[29], [33]. We took advantage of the knowledge developed for PBMC isolation and processing by these other fields to explore factors that could impact these cells, while recognizing that the factors, which impact functional cytokine release assays with PBMCs, may be different than those for more straightforward SMN protein analysis. The experience in these other research areas suggested the need to generate a protocol for optimized processing for the purpose of SMN protein analysis. The development of such a protocol was attempted through a series of healthy adult, carrier and SMA patient sample analyses. PBMCs are sensitive to external stimuli and we found that SMN signal in these cells are modulated by collection and processing methods, storage conditions, extraction and lysis reagents, infection status and possibly age [27]–[29], [33]–[36]. While SMN protein from human samples has been measured for over a decade by researchers in the SMA community and multiple assays exist for evaluating concentration of this protein in PBMCs, the generation of this SMN PBMC protocol is unique in the extent of variables examined [8], [22], [23], [25], [37]. It is important to note that Ns were small in our studies, and subsequent followup with larger samples would be helpful.

An optimized protocol for protein signals in PBMCs for a multisite clinical trial would ideally require minimal processing site by site and analysis by a central site to reduce variability. Our studies began using a protocol very similar to the one recommended by the Immune Tolerance Network, and used in the Biomarkers for SMA Study Group [2], [38]. PBMCs collected by CPT tubes were susceptible to reduction in cell yields and delays in processing of up to 24 h either before or after cell isolation resulted in dramatic loss of SMN signal and triggered increases in total protein levels (Figures 1–2). In contrast, collection of whole blood by EDTA tubes and cell isolation by Lymphoprep resin gradients produced samples with higher PBMC counts and no apparent alterations in total protein content with up to 24 h processing delays, similar to prior reports in the HIV field [26] (Figure 3). These data from Lymphoprep PBMC isolation are consistent across timepoints and enable researchers to collect blood samples and isolate PBMCs at multiple sites and ship them overnight to a central facility for more uniform processing to lysates. Moreover, the SMN protein signal in SMA patients and carriers appears to be stable with up to 6 months of storage at −80°C, allowing for more cost-effective and infrequent sample analysis in longitudinal studies (Figure 8D). It is notable that freezing and thawing isolated PBMCs collected by CPT tubes in these experiments caused cell recovery and viability to plummet (Figure 2A–B). Freezing PBMCs for later use in functional cytokine assays and viral burden assays is routinely done in other fields, in some cases with stability of results over 10 years in cold storage [28], [33]. Further work focused on the optimization of freezing media, DNAse treatment after cell thawing, reductions of pre-freezing time and use of −140°C storage could improve the ability to recover PBMCs for subsequent SMN processing, though it would give rise to protocols requiring more extensive sample processing and special equipment [39].

Reagent evaluations were helpful in producing strong SMN signals in PBMC samples. Previous work with the same SMN ELISA used in this study showed dramatic differences in SMN levels across mouse tissues [37], [40], and analysis of whole blood and red blood cell pellets indicated that red blood cell (RBC) populations interfered with signal detection and/or had lower total signals than PBMCs (Table 1). Additive increases of >2x in SMN PBMC signal was observed when removing contaminating red blood cells from the sample and using PBMC lysates with 10^7^ cells/mL ER4 (Figure 4A, B). While SMN was readily detectable in samples containing RBCs, interference in this matrix was revealed by simple dilution (Table 1). While other factors that alter or improve SMN signal in RBCs and whole blood were not examined, this remains an area for additional investigation.

Even with protocols aimed at reducing sample variability and instability and improving signal detection, PBMCs remain a matrix inherently sensitive to a number of stimuli *in vivo,* and signal normalization is an important consideration. Results generated by total protein and PBMC count as normalization factors generally agreed with each other, though it was clear that these factors were differentially affected by processing delays (Figure 1D–E, 2D–E). It is recommended that both normalization measures be used wherever possible and that efforts be taken to ascertain whether novel SMA drugs in trials themselves have impact on PBMC count and total protein levels. Studies 5 and 6 focusing on SMN levels across timepoints in the same individuals revealed substantial fluctuation in signals and emphasized the impact of respiratory infections on PBMC subpopulations, particularly CD14+ cells involved in innate immunity responses (Figures 6D–E,7). While the sample sizes from these experiments was small and serve to illustrate the possible sources impacting SMN measurements in PBMCs, the seemingly modest variability in the CD8+ population merits further analysis in a larger study to explore whether it may indeed be useful for SMN signal normalization, possibly by cell sorter analysis. Cell sorting may be important because the CD+ cell populations quantified in Study 4 only represent 40–70% of the total PBMC fraction and other unmeasured cell types could figure heavily into the overall SMN analysis. Other efforts to reduce technical variability and improve precision could center on automating certain aspects of the process, e.g. use of automated cells counting devices. Normalization to DNA content in cells also represents an alternative to normalization to protein content (which seemed to be sensitive to processing conditions (Figure 1C, 2C). However, it must be mentioned that others have reported meaningful differences in DNA content depending on the extraction method used, and so careful optimization for the methods would also be required [41].

The variability observed in relation to different factors underscored the need to collect additional medical information to better interpret SMN protein results in PBMCs. While there was no clear effect on the 6 h timepoint from eating a large pasta meal in Study 5, carbohydrate-rich meals have been reported to stimulate PBMCs [35]. In SMA trials it is impractical to implement fasting or diet controls on a fragile pediatric population, though it would be generally useful to standardize the timing of blood collections across subjects and collect information on recent nutritional intake. In Study 6, PBMC subpopulations were dramatically altered at t = 0 h during symptomatic respiratory infection and SMN signal in CD14+ was notably low (Figure 7). In particular, one subject (1036) had very high levels of SMN following a period of recurrent respiratory infections and/or allergies, and without further clinical history information is unclear if chronic SMN elevation typically follows chronic infection or allergies or if this is a precursor to new episode of respiratory symptoms.

Study SMAF-001 is one of the first to examine SMN signal in SMA patients and carriers using a commercially available SMN quantification kit and protocol optimized for PBMCs. SMN signals varied greatly and were indistinguishable by Type among the patient and also carrier subjects (Figure 8A). These limited data recapitulate the premise that SMN protein levels themselves are not reliable for diagnosing SMA, though copy number is generally related to disease severity [2], [8], [42], [43]. Although the sample size was small, there was tendency for SMN protein signal to be greater in samples from patients with higher current motor function and is a topic for further analysis with larger collections (Figure 8B, Table 3).

Age seemed to be an important covariate with respect to SMN levels in SMAF-001, as it was in the larger Biomarkers for SMA Study which showed SMN transcript was lower over age in children 2–12 years old [2]. PBMCs have been reported to be more numerous in children compared to adults, and this finding was replicated in this study (Figure S2) [36]. SMN protein levels in PBMCs in SMA patients and carriers appeared to be lower with increasing age, and when controlling for age SMA patients had significantly different SMN levels from adult carriers (Figure 8C). It is intriguing to speculate that this result in blood cells is similar to reports that show SMN decreases through development in brain and spinal cord tissues in SMA model mice, since this relationship would support use of PBMCs as a surrogate tissue for SMN upregulation drug trials [37], [40]. However, to confirm the value of PBMC SMN as a pharmacodynamic marker for SMA, these data must be reproduced in a prospective study with greater sample numbers, with healthy pediatric subjects if possible. Moreover, the confirmation study in SMA patients should include reasonable sample numbers in selected age groups as they may experience increases, decreases, or stability in motor function at different points in time [44].

In SMAF-001 there were a number of subjects taking drugs that have putative effects on SMN levels or have side effects on blood cells [20], [45], [46]. Given the sample size and single visit format of the study, it is unclear whether valproic acid and/or phenylbutyrate had any positive effects on SMN levels (Figure 8C, arrows): patients younger than 4 years had higher levels of SMN but half of these subjects were taking putative SMN-upregulating drugs. Some drugs being taken by the patients includes some with known effects on white blood cell count (Atenolol, gabapentin, phenylbutyrate), or otherwise effect blood components like platelets (valproic acid). Though there is no clear causal relationship between medication and difficulties in PBMC processing or lower cell counts, it is nonetheless important to know and attend to the possibility of drugs affecting PBMCs and the interpretation of SMN signals in SMA trials.

While this report details a number of studies focused on optimizing protocols for SMN protein analysis in PBMCs, the same principles for optimization likely will apply to SMN transcript measures. The concept that sample matrix processing protocols must be rigorously optimized for each particular use and analyte within the sample is exemplified by our own experiences: the PBMC harvest and isolation protocol from the Immune Tolerance Network that is highly effective for enabling cytokine release assays was not optimal in our hands for SMN protein analysis. Candidate methods for SMN transcript quantitation (including an absolute quantification method), have been published [21], [24], [47]–[49]. The relative importance of any particular factor that affects PBMC processing for protein versus nucleic acid may be distinct depending on the analyte in question, and there is literature to suggest that some RNA signals may be differentially sensitive to processing delays [34], [50]. As a guiding principle, rapidly processing samples of any type often yields the best results, and thus future efforts to optimize methods for SMN transcript in blood samples may necessarily impact SMN protein measures in PBMCs. While it is possible to process PBMCs for SMN protein reliably with up to 24 h delays in cell isolation, it is recommended that PBMCs be separated within 2 h for best results. With this in mind, the optimized standard operating protocol for measuring SMN protein in PBMCs by ELISA is available online for further exploration by the field: http://www.treat-nmd.eu/downloads/file/sops/sma/SMA_M.1.2.005.pdf.

The collective studies reported here were done in anticipation of SMA trials that will use PBMCs as a matrix for measuring changes in SMN protein. PBMCs are a sensitive matrix and our experiments show how variable the cells and SMN protein measures can be in response to processing delays prior to the generation of stable cell lysates. Optimization of PBMC processing methods enables measurement of SMN in a manner compatible with clinical trials at multiple sites, and other normalization methods may be of additional value in reducing variability. These studies suggest that it is important to interpret the SMN data in PBMCs in the context of medical history related to recent systemic infections and/or any concomitant medications, as both of these factors could impact the signal of interest. Furthermore, it is important to point out that the protocol developed through these experiments permit sample analysis with pediatric subjects and that 1 mL of whole blood is expected to be sufficient to provide multiple aliquots for PBMC SMN analysis. In future experiments it would be valuable to determine in human tissues whether SMN in PBMCs can provide readout on SMN in target tissues in the spinal cord and elsewhere. This additional data is critically important as it would confirm SMN in PBMCs as a biomarker for target engagement and perhaps dosing modulation in SMA. Finally, it is of general community interest to determine whether SMN in PBMCs and other more disease-relevant cells does indeed decrease longitudinally, as it would help guide therapeutic expectations on whether SMN-upregulating drugs could arrest or reverse motor decline over time.

## Materials and Methods

### Ethics Statement

Healthy adult and SMA patient samples from the Jasper Clinic (Kalamazoo, MI) were collected in accordance with study protocols approved by the Bronson Methodist Hospital Institutional Review Board (IRB) (Kalamazoo, MI), with written consent from adult subjects and patient parents, and assents for children over 7 years of age. Healthy adults were recruited by the Jasper Clinic, while SMA patients and carriers were notified of the study via direct mail and email communications with the local Families of SMA chapter. Samples from AllCells (Emeryville, CA) were collected from adults who gave written consent in accordance with protocols approved by their governing IRB (Biomed).

### PBMC Sources and Processing Summary

Healthy adult PBMCs were collected from N = 11 individuals at the Jasper Clinic for analysis in 6 separate studies; some individuals participated in multiple studies. Subjects were non-fasting, non-smoking adults 18–35 years of age, who were not on medications for blood disorders, and had not given blood for one month prior to study. PBMCs from SMA carriers (N = 15) and SMA patients (N = 12) were collected, processed and analyzed for Study SMAF-001 at the Jasper Clinic. No genotyping was performed on any individuals in these studies, and all subjects self-identified themselves as SMA patients and carriers based on prior testing and diagnoses. For reagent concentration and lysis experiments healthy adult PBMCs were purchased from AllCells (Emeryville, CA).

Whole blood samples at the Jasper Clinic were collected in BD CPT tubes (#362761, Studies 1–3), EDTA tubes (#22-040-67, Fisher, Pittsburgh, PA in Studies 3–5, and SMAF-001) or ACD tubes (#363083, BD, Franklin Lakes, NJ in Study 5). PBMCs were processed at PharmOptima (Portage, MI) and isolated by CPT tubes (Studies 1–3) or by Lymphoprep gradients (#1114545, Accurate Chemical, Westbury, NY) Studies 3–5 and SMAF-001).

### PBMC Fractionation Analysis

Briefly, PBMCs isolated from each subject were suspended in 10 mL of wash buffer (98% Dulbecco’s’ PBS (without Ca^2+^ and Mg^2+^ containing 2% Fetal Bovine Serum) and centrifuged at 300×g for 5 minutes at room temperature. The supernatant was aspirated and the cells suspended in 0.3 ml of wash buffer and 50 µl of Miltenyi bead solution added to the cells followed by the addition of 100 µl of fresh wash buffer. The order of addition of magnetic beads with antibodies for positive selection of PBMC subsets were CD14, CD56, CD19, CD8, and CD4 (Miltenyi OctoMACs #130-050-201, #130-050-401, #130-050-301, #130-045-201, #130-045-101 respectively). Cells were incubated for 15 minutes at room temperature and then 9.5 ml of wash buffer added to the cells and the cells centrifuged for 5 minutes at 300×g at room temperature. The supernatant was aspirated and 500 µl of fresh wash buffer added to the cells. Each magnetic separation (MS) column was washed with 500 µl of wash buffer and then the 500 µl of cells added to the MS column on the magnet. The cells flowing through the column were collected as the “negative” fraction for a given selective antibody and used for subsequent isolation of other cell subsets. The solution dripping through the column was allowed to completely drip through before washing the column with 2.0 ml of wash buffer. Each column was washed a second time with 2.0 ml of wash buffer and the solution allowed to completely drip through the column. The column was removed from the magnet and the “positively” selected cells washed off the column and collected by adding 3.2 ml of wash buffer to the column and immediately taking the plunger and forcing the liquid through the column. Positively selected cells were counted and viability assessed and the cells adjusted to 10^7^ viable cells per ml of ER4 buffer for lysis. The supernatant was centrifuged at 14,000×g to clarify the lysate and the lysate frozen and stored at −80°C.

### Cell Counting and Lysis

For cell counting, a 25 µL aliquot of each cell suspension was added to 75 µL of tryptan blue (1∶4 dilution). Within 5 minutes of addition to tryptan blue, the cells were counted using a hemocytometer. Viability, viable cells per mL and the total live cells were calculated. After counting, the PBMC were pelleted by centrifugation at 300×g at room temperature for 15 minutes. The supernatant was discarded and ER4 lysis buffer (Enzo Life Sciences, Farmingdale, NY) containing PMSF (#P7626, Sigma) and protease cocktail inhibitors (#P8340, Sigma) added to the pellet at a ratio of 100 µL per million viable cells (1×10^7^ cells per mL). The pellet was pipetted up and down 3 to 4 times to ensure a homogenous suspension of cells into lysis buffer. Each tube was then vortexed for ∼15 seconds and left on ice for 30 minutes. At the end of the 30 minute incubation, each sample was vortexed for approximately 15 seconds just prior to centrifugation at 3200×g at 4°C for 15 minutes. The resulting clear supernatant was transferred to cryovials in ∼100 µL aliquots then frozen on dry ice until storage at −80°C.

### SMN Protein Quantitation

On the day of SMN analysis an aliquot of the PBMC lysate was analyzed for soluble protein content using a colorimetric commercial kit and following manufacturer instructions. Standards were prepared in 20% ER4 lysis buffer diluted in distilled water. SMN protein was quantified by ELISA (#ADI-900-209, Enzo Life Sciences, Farmingdale, NY). Each lysate was also diluted in assay kit buffer containing 1∶4 or 1∶8 ER4 except for the optimization study that examined SMN signal in various dilutions of assay buffer. Each of these dilutions was serially diluted 1∶2 into 20% ER4 in assay kit buffer. Standards were prepared in 20% ER4 in assay kit buffer ranging from 3200 to 25 pg/mL. Quality control samples were diluted in 20% ER4 in assay kit buffer at 3 concentrations. All samples and standards were assayed using 100 µL per well in duplicate.

### DNA Quantitation

After counting, PBMCs were serially diluted 1∶1 in PBS. A 10 µl sample of cells in PBS was diluted 1∶10 with Hanks Balanced Salt Solution (HBSS) provided in a DNA quantification kit (Cyquant #35007), and 50 µl of cells added to a black 96-well black culture plate. A 50 µl aliquot of 2x DNA binding dye was added to the cells and the cells incubated for 30 minutes at 37°C. Following the 30 minute incubation, the plate was read using a fluorescence plate reader at 485 nm excitation and the 520 nm emission wavelengths.

### Analysis of Lysis Buffer Concentration, Erythrocyte Buffer Treatment, and Assay Buffer on SMN Signal

A bulk aliquot of 5×10^7^ frozen normal PBMCs was thawed and washed according to a protocol provided by AllCells (#PB004F). Samples were counted and evaluated for total soluble protein levels prior to treatment in ice-cold erythrocyte lysis (EL) buffer (Qiagen #79217) or PBS for 5 minutes at room temperature with subsequent centrifugation at 1500rpm for 20 min and resuspension into 5 mL PBS prior to cell counting. Lysis of EL or PBS-treated PBMCs was performed in 10^6^, 10^7^ or 50^7^ cells/mL concentrations of ER4 reagent. SMN protein signal was quantified in lysates diluted at 1∶2, 1∶4, 1∶8, 1∶16 and 1∶32. An additional thawed PBMC sample was assessed for SMN signal spike recovery against 200, 800, and 1600pg/mL of recombinant SMN protein in the ELISA. The spike recovery was assessed using different assay dilutions of ER4 buffer, from neat, 1∶2, 1∶4, 1∶8, and 1∶16.

### Study 1: Impact of Short-term PBMC Isolation Delays

Blood was drawn into 4 mL sodium citrate BD-CPT tubes with a 21G needle. A total of 12 samples (4 per patient) of whole blood were drawn from 3 healthy individual subjects (2 males, 1 female) at the same time (approximately 8∶40 AM). All blood samples were mixed by inversion 10 times immediately after drawing, then kept upright at room temperature until centrifugation. Either immediately, 45 minutes, 2 h, or 24 h after the blood draw, 1 CPT tube per patient was mixed by inversion 10 times, then centrifuged 1800×g at room temperature for 30 minutes with the brake off. Following centrifugation three distinct bands were present in each CPT tube: the top plasma layer was above the gel separator which was above the RBC’s and neutrophils. Using a sterile transfer pipette the top half of the plasma layer was removed and discarded. The stopper was placed back into the CPT tube and it was mixed gently by inversion 2–3 times. The remaining plasma layer which contained the PBMC was transferred using a sterile pipette to a clean 15 mL tube. The PBMCs were washed by the addition of ∼13 mL PBS and mixed by inversion. The PBMC were then pelleted by centrifugation at 300×g at room temperature for 15 minutes. The supernatant was discarded and 1 mL of PBS added using a1 mL pipette. The PBS was pipetted up and down 2 to 3 times to gently mix the cells. The final volume of each tube was adjusted to 4 mL. Cells were lysed at 10^7^ cells/mL ER4 on ice for 30 minutes, and prepared for SMN ELISA analysis or frozen storage at −80°C. Lysates were diluted 1∶5, 1∶10 and 1∶20 in 20% ER4 for analysis.

### Study 2: Impact of Post-isolation Delay and Cell Freezing

Study 2 was performed similarly to Study 1 with a few exceptions. In Study 2 blood was collected from 4 healthy volunteers and PBMCs were isolated 1 hr after collections. Sample aliquots were created and were processed to fresh PBMC lysates or frozen as cells immediately, 45 minutes, 2 h or 24 h after PBMC isolation. Half of the PBMCs were mixed with Sigma cell-freeze medium (#C6164, St. Louis, MO) and frozen overnight in a Mr. Frosty container with 2-propanol (#5100–0001, Nalgene, Rochester, NY) at −80°C after the appropriate post-processing delay. The other half of the PBMCs were processed to lysates after the appropriate post-processing delay and frozen at −80°C until further analysis. After 24 h at −80°C, the frozen PBMCs were thawed and lysed. Thawed lysates were analyzed for total soluble protein levels prior to testing in the ELISA as in Study 1. SMN protein levels were then measured at the various post-isolation time points with the fresh lysates as well as the lysates generated from frozen cells with dilutions similar to Study 1.

### Study 3: Comparison of PBMC Isolation Methods and SMN Measures from Various Blood Fractions

A single 4 mL BD-CPT sodium citrate tube was drawn from each patient using a 21G needle, for a total of 4 samples. Time 0 was designated as the time of blood draw. Samples collected into CPT tubes were processed as in Studies 1 and 2 with a 45 min delay prior to centrifugation. Also, using 6 mL K_2_EDTA tubes, an additional 4 tubes per patient were drawn, for a total of 16 samples. The samples collected into K_2_EDTA tubes were then split and transferred immediately (0), at 2 h or 24 h, to either a CPT tube containing citrate, or diluted 1∶1 with PBS and placed into a tube containing 3 mL Lymphoprep solution for density gradient centrifugation. Lymphoprep tubes were briefly centrifuged at 800×g at room temperature for 15 minutes with the brake off. Following centrifugation a distinct white band above the frit was transferred using a sterile pipette to a clean 15 mL tube for counting.

Approximately 2 h after collection of blood, one of the 6 mL K_2_EDTA tubes from each patient was used for whole blood processing. K_2_EDTA tubes were inverted 10 times to mix just prior to removal of 0.5 mL whole blood. The remaining whole blood was centrifuged at 4000 rpm at room temperature for 10 minutes. The plasma layer was discarded and the blood pellet was processed further. To the whole blood and blood pellet samples, ER4 lysis buffer containing protease inhibitors was added (0.5 and 1.0 mL, respectively) as processed as described in Studies 1 and 2. The supernatants (red in color) were saved for SMN analysis.

### Study 4: Intraindividual SMN Signal Variability Over Time

Healthy adult blood was collected from 6 subjects in 4 ml K_2_ EDTA tubes at time 0 (8∶30–9∶30 AM), and again collected at 6 h, 24 h, 7 days and 30 days. Information about subject age, gender, cold symptoms, onset, was collected at the first timepoint, and information about medications taken for the past 2 weeks was collected at the t = 0, 7, and 30 day timepoints. Subjects ate normally the night before initial sample collection and three hours after the t = 0 timepoint, subjects were fed a 786 calorie pasta meal comprised of 69% carbohydrates, 15% protein, and 21% fat, so that blood collected at the 6 h timepoint was 3 h post-prandial. The blood was stored at room temperature overnight at PharmOptima and processed the following day to isolate PBMCs. The PBMCs from whole blood was isolated similar to the K_2_ EDTA collection group in Study 3, using a Lymphoprep gradient followed by a 5 minute treatment with erythrocyte lysis buffer to eliminate red blood cell contamination. PBMCs were adjusted to 10^7^ cells per ml before lysis of the cells in ER4 buffer reagent, and PBMC lysate was quantitated for SMN within a week of sample collection with the SMN ELISA kit as in prior studies. For the day 7 sample collection PBMCs were also fractionated into T cells, natural killer cells, monocytes, and B cells using Miltenyi magnetic bead columns (Auburn, CA). Cell subsets were positively selected using microbeads for CD8 for cytotoxic T-cells (#130-045-201), CD4 T helper cells (#130-045-101), CD19 B cells (#130-050-301), CD56 natural killer cells (#130-050-401), and CD14 monocytes (#130-050-201); the cellular subsets were evaluated individually for SMN protein. All SMN analyses with total PBMC and PBMC subtypes were performed at 1∶2, 1∶4, 1∶8, and 1∶16 dilutions.

### Study 5: Immune Modulation of PBMC SMN Signal

Blood was collected from 2 subjects at a time when they were experiencing the signs and symptoms of a cold, with subsequent collections at 7, 30, and 76 days after the initial sampling. Information about the subject’s age, gender, cold symptoms, onset, was collected at the first timepoint. Blood was collected in 4 ml EDTA tubes at time 0 (8∶30–9∶30 AM). Samples were processed as described for Study 4, and samples from the 7 day timepoint were also subjected to cell subtype fractionation using the Miltenyi microbead system. PBMC lysate will be quantitated for SMN within a week of sample collection using the SMN ELISA, and all PBMC lysates will were also evaluated a second time approximately 30 days after sample collection from lysates stored at −80°C.

### SMAF-001: Measurement of SMN Protein in SMA Patient and Carrier PBMCs

Blood from SMA carriers and patients was collected during a single clinic visit and processed as described in Study 4 for analysis of SMN protein in PBMC lysates at 48 h, 1 month, 3 months and 6 months after sampling. Information about gender, age, SMA or Carrier status, genetic relationship to any enrolled SMA patient (parent, sibling), SMN2 copy number, SMA Type, age at onset, Hammersmith Functional Motor Scale (HFMS) or equivalent, patient’s current level of function (ambulatory or non-ambulatory), current or highest motor function achieved (sitting with assistance, sitting, rolling, crawling, standing, walking with assistance, walking) as well as information on any medications taken in the past 7 days, including any putative SMN-enhancing or palliative drugs used in SMA studies (e.g. valproic acid, phenylbutyrate, hydroxyurea, carnitine, riluzole, creatine, oral albuterol). Medications for the SMA patients included putative SMN upregulating drugs (N = 3 with valproic acid, N = 1 with valproic acid and with one sodium phenyl butyrate) as well as several other prescription drugs like al (N = 1), Atenolol (N = 1), bethanechol (N = 1), gabapentin (N = 1), Flonase (N = 1), Glucophage/metformin (N = 2), Lipitor/atorvastatin (N = 2), Prilosec/omeprazole (N = 2), Singulair (N = 2), Xanax/alprozalam (N = 2), and Zyrtec (N = 1). Blood volumes of 3–11 mL were collected for SMA subjects and 6–9.5 mL for Carriers.

### Optimized PBMC Processing Protocol

PBMC’s were isolated from whole blood collected in EDTA blood collection tubes and the PBMC’s isolated by density gradient centrifugation using Lymphoprep. Blood samples were diluted 1∶2 using room temperature PBS. Diluted blood was then layered onto 3 ml of Lymphoprep solution in a 15 ml centrifuge tube. The layered sample was then centrifuged at 2500 rpm at 20°C for 20 minutes with the brake off. Following centrifugation PBMC’s were harvested from the interface by pipetting the buffy coat into a separate 15 mL centrifuge tube. Ice cold PBS was then added to a final volume of 15 mL and mixed by inversion. Following mixing cells were collected by centrifugation at 1500 rpm in a Beckman CS-6R centrifuge at 4°C for 10 minutes with the brake on low. Supernatant was removed by aspiration and cells were re-suspended in 5 mL of ice cold erythrocyte lysis buffer (EL) and incubated on ice for 5 minutes. Following incubation cells were collected by centrifugation in a Beckman CS-6R centrifuge at 1500 rpm set at 4°C for 10 minutes with the brake on low. The cells were re-suspended in 5–10 ml of PBS and enumerated by direct microscopic count using a hemocytometer. Cell viability was monitored by tryptan blue exclusion. Cells were then collected by centrifugation in a Beckman CS-6R centrifuge at 1500 rpm for 10 minutes at 4°C with the brake on low, and the cells lysed using Extraction Reagent 4 (ER4 supplied with the SMN ELISA kit) containing protease inhibitors at a final cell density of 10^7^ cells/mL. Lysates were clarified by centrifugation at 14,000 rpm and clarified lysates were frozen and maintained at −80°C until the time of assay.

### Statistical Methods

Analysis of statistical significance between SMN levels in between groups was done by one-way ANOVA with Tukey’s test or an unpaired t-test using Prism software by GraphPad (La Jolla, CA). Correlation analyses e.g. for association between Subject Age and SMN protein levels or SMN levels at different timepoints in SMAF-001 were performed using Pearson’s test. P-values are indicated by asterisks or plus signs in the following manner: p<0.001 by ***, p<0.01 by ** and p<0.05 by *. In figures error bars depicted are standard deviations except where specified otherwise.

## Supporting Information

Figure S1 **PBMC SMN levels in individual subjects with different sample collection methods and processing delays.** Data from four subjects with different PBMC collection methods were displayed to depict SMN changes over time on an individual basis. A: SMN protein normalized by PBMC count for EDTA/CPT processed samples. B: SMN protein normalized by PBMC count for EDTA/Lymphoprep processed samples. C: SMN protein normalized by soluble total protein for EDTA/CPT processed samples. D: SMN protein normalized by soluble total protein for EDTA/Lymphoprep processed samples. The same individuals are represented with the same shapes across all panel, whether with filled or open shapes.
(TIF)

Figure S2 **SMN in SMA patients and Carriers normalized by PBMC count.** SMN protein (normalized by PBMC count) was evaluated in Type 2 and 3 SMA patients and Carriers for differences by Type and motor function. **A**: SMN protein classified by Type and Carrier status. **B**: SMN levels differentiated by current motor function appeared to distinguish between sitters and ambulatory patients.
(TIF)

Figure S3 **PBMC counts in SMA patients and Carriers.** PBMCs were counted by a hemocytometer. Total PBMCs isolated and collected blood volumes ranged from 0.8–70×10^6^/mL and 3–11 mL for SMA patients and 4.8–17.6×10^6^/mL and 6–9.5 mL for Carriers. ** = p<0.01.
(TIF)

Table S1 **PBMC counts in subjects with respiratory infection in Study 5.** All PBMC subtypes are represented as 10^6^ cells. Values depicted with % signs represent the percentage of a particular subtype cells among the total PBMC cell count for that timepoint.
(DOCX)
