# Supplementary figures and images for: Evaluation of Peripheral Blood Mononuclear Cell Processing and Analysis for Survival Motor Neuron Protein
Source: PLoS One. 2012 Nov 30;7(11):e50763. doi: 10.1371/journal.pone.0050763 (PMC3511312; doi:10.1371/journal.pone.0050763)

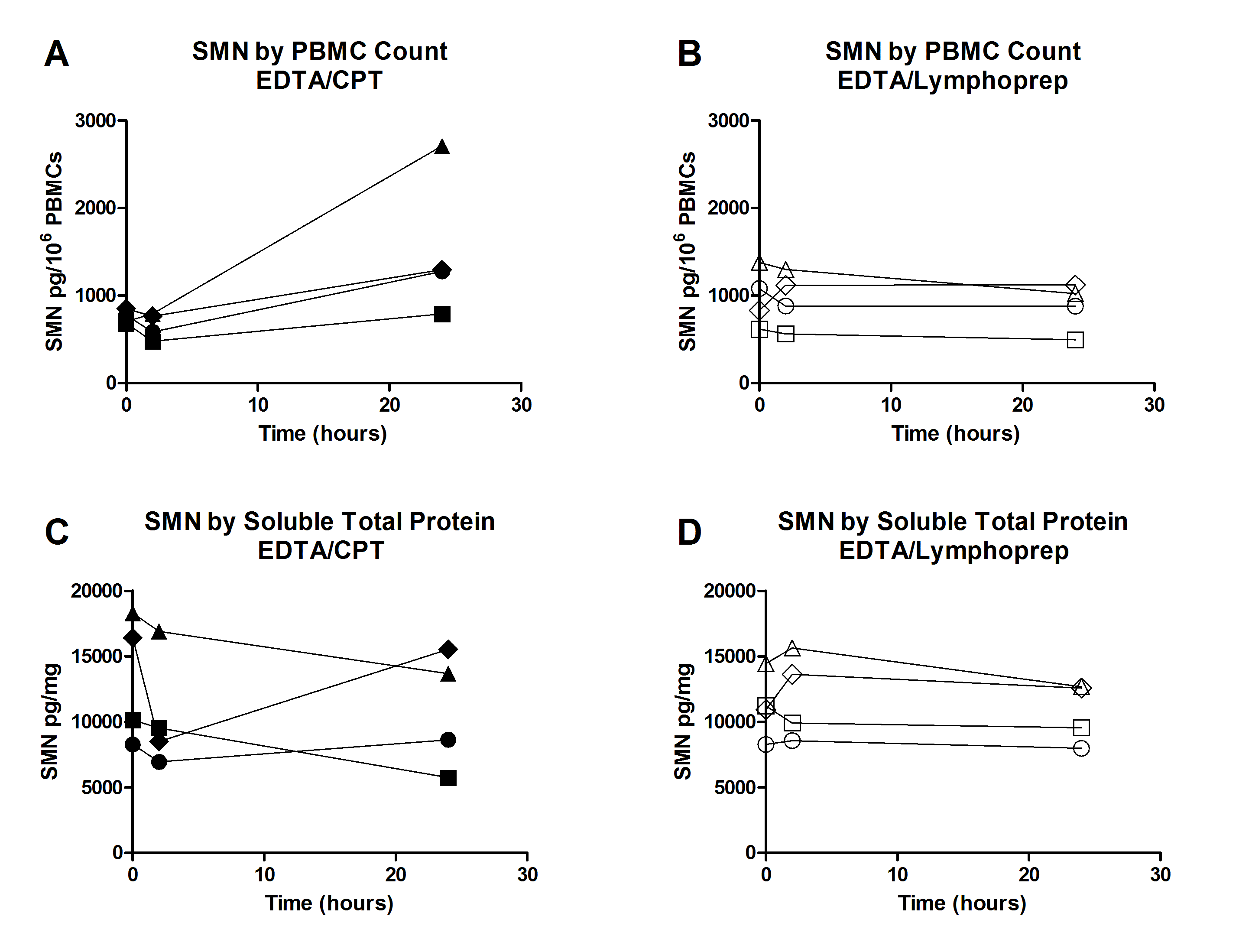

Supplement: Figure S1 — PBMC SMN levels in individual subjects with different sample collection methods and processing delays. Data from four subjects with different PBMC collection methods were displayed to depict SMN changes over time on an individual basis. A: SMN protein normalized by PBMC count for EDTA/CPT processed samples. B: SMN protein normalized by PBMC count for EDTA/Lymphoprep processed samples. C: SMN protein normalized by soluble total protein for EDTA/CPT processed samples. D: SMN protein normalized by soluble total protein for EDTA/Lymphoprep processed samples. The same individuals are represented with the same shapes across all panel, whether with filled or open shapes. (TIF) [file pone.0050763.s001.tif]

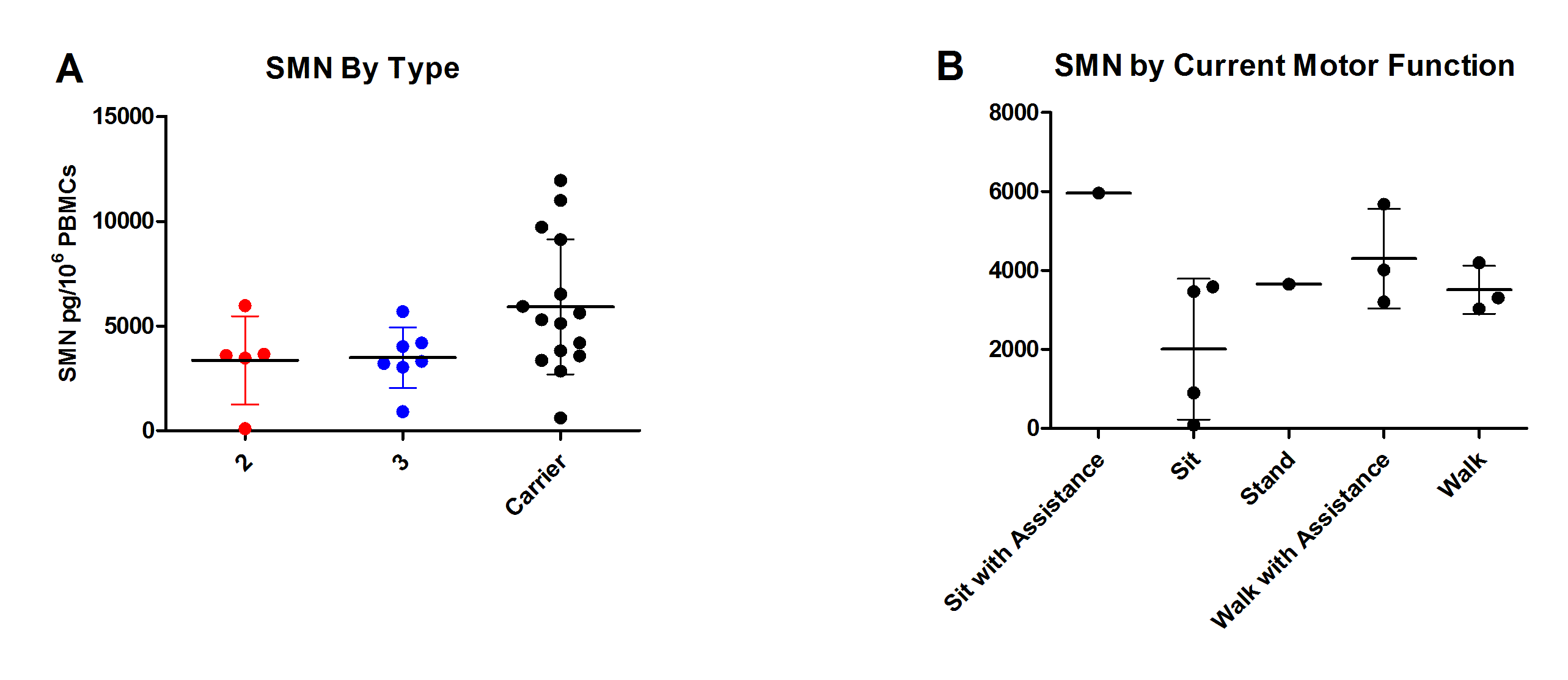

Supplement: Figure S2 — SMN in SMA patients and Carriers normalized by PBMC count. SMN protein (normalized by PBMC count) was evaluated in Type 2 and 3 SMA patients and Carriers for differences by Type and motor function. A: SMN protein classified by Type and Carrier status. B: SMN levels differentiated by current motor function appeared to distinguish between sitters and ambulatory patients. (TIF) [file pone.0050763.s002.tif]

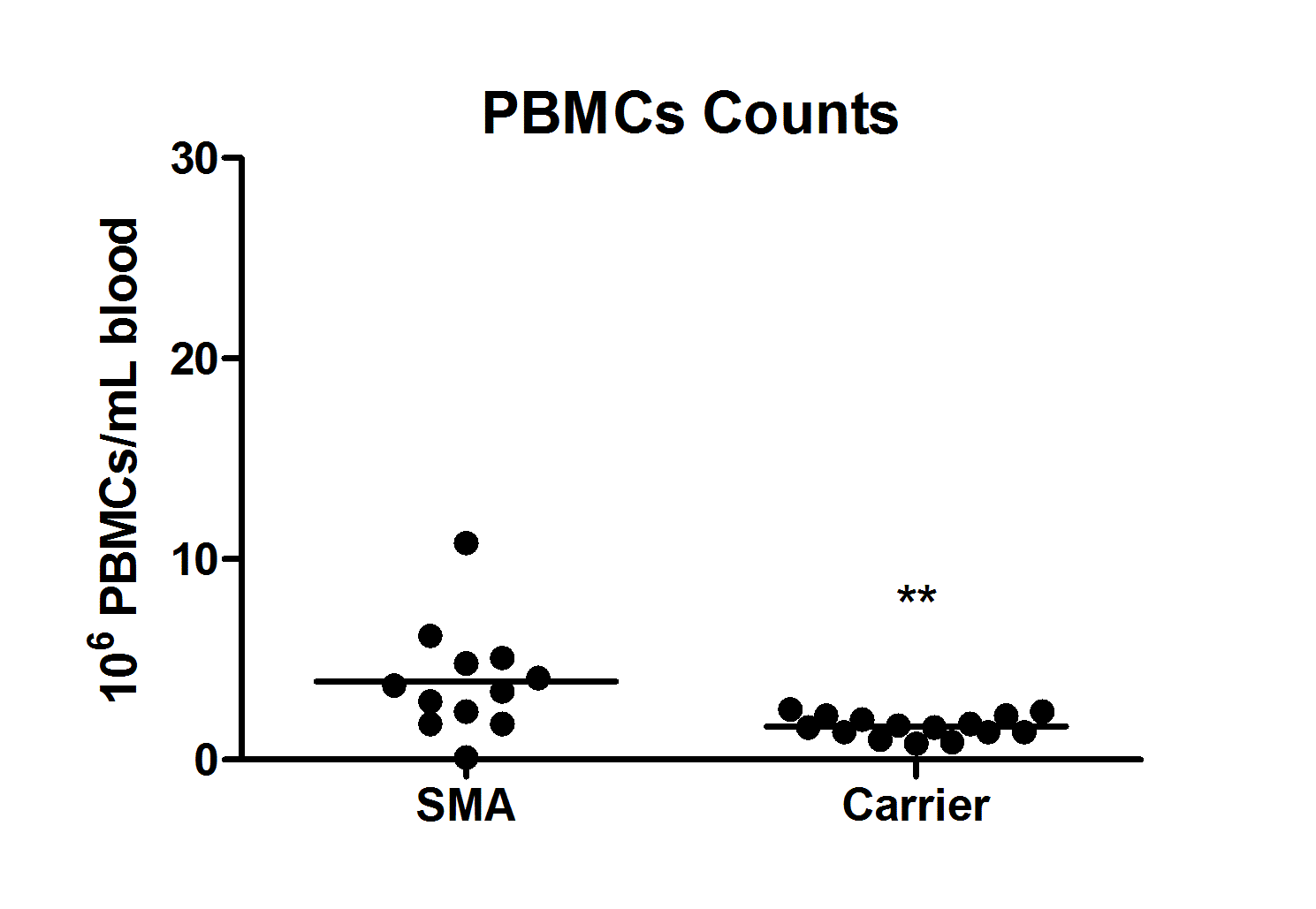

Supplement: Figure S3 — PBMC counts in SMA patients and Carriers. PBMCs were counted by a hemocytometer. Total PBMCs isolated and collected blood volumes ranged from 0.8–70×106/mL and 3–11 mL for SMA patients and 4.8–17.6×106/mL and 6–9.5 mL for Carriers. ** = p<0.01. (TIF) [file pone.0050763.s003.tif]
