# Supplementary material for: Evaluation of Peripheral Blood Mononuclear Cell Processing and Analysis for Survival Motor Neuron Protein
Source: PLoS One. 2012 Nov 30;7(11):e50763. doi: 10.1371/journal.pone.0050763 (PMC3511312; doi:10.1371/journal.pone.0050763)
Supplement: Table S1 — PBMC counts in subjects with respiratory infection in Study 5. All PBMC subtypes are represented as 106 cells. Values depicted with % signs represent the percentage of a particular subtype cells among the total PBMC cell count for that timepoint. (DOCX) [file pone.0050763.s004.docx]

Evaluation of Peripheral Blood Mononuclear Cell Processing and Analysis for Survival Motor Neuron Protein

**Dione T. Kobayashi PhD^*^, Douglas Decker, Phillip Zaworski, Karen Klott, Julie McGonagall, Nabil Ghazal PhD, Laurel Sly, Brett Chung, James Vanderlugt MD, Karen S. Chen PhD**

From the Spinal Muscular Atrophy Foundation (DTK, BC, KSC) New York, NY, USA; PharmOptima LLC. (DD, PZ, LS) Portage, MI, USA; Jasper Clinic, Kalamazoo, MI (KK, JM, NG, JV)

*Corresponding author: Dione T. Kobayashi (dkobayashi@smafoundation.org)

### Supplementary Table S1. PBMC counts in subjects with respiratory infection in Study 5

| Subject | Timepoint | Total PBMCs (10^6^) | CD14+ | CD56+ | CD19+ | CD8+ | CD4+ |
| --- | --- | --- | --- | --- | --- | --- | --- |
| 1002 | Day 0 | 52.2 | 2.3 (4.4%) | 2.4 (4.6%) | 2.9 (5.5%) | 4.3 (8.2%) | 7.9 (15.2%) |
|  | Day 7 | 44.8 | 3.5 (7.8%) | 1.6 (3.5%) | 3.0 (6.7%) | 5.2 (11.5%) | 6.6 (14.7%) |
|  | Day 30 | 44.8 | 2.0 (4.4%) | 0.9 (2.0%) | 1.6 (3.5%) | 3.6 (7.9%) | 5.8 (12.9%) |
|  | Day 76 | 27.6 | 3.9 (14.1%) | 0.7 (2.5%) | 1.6 (5.7%) | 1.4 (4.9%) | 1.9 (6.7%) |
|  |  |  |  |  |  |  |  |
| 1036 | Day 0 | 32.2 | 2.7 (8.4%) | 2.3 (7.2%) | 1.4 (4.4%) | 3.4 (10.6%) | 6.6 (20.6%) |
|  | Day 7 | 35.6 | 3.4 (9.6%) | 2.2 (6.1%) | 1.7 (4.7%) | 4.4 (12.3%) | 5.6 (15.8%) |
|  | Day 30 | 32.4 | 3.0 (9.2%) | 1.9 (5.8%) | 1.4 (4.4%) | 3.6 (11.2%) | 5.4 (16.5%) |
|  | Day 76 | 31.6 | 2.9 (9.1%) | 3.3 (10.4%) | 1.6 (5.2%) | 2.4 (7.5%) | 3.0 (9.6%) |

All PBMC subtypes are represented as 10^6^ cells. Values depicted with % signs represent the percentage of a particular subtype cells among the total PBMC cell count for that timepoint.
